# Role of the D1-D2 Linker of Human VCP/p97 in the Asymmetry and ATPase Activity of the D1-domain

**DOI:** 10.1038/srep20037

**Published:** 2016-01-28

**Authors:** Wai Kwan Tang, Di Xia

**Affiliations:** 1 Laboratory of Cell Biology, Center for Cancer Research, National Cancer Institute, National Institutes of Health, Bethesda, MD 20892, USA

## Abstract

Human AAA^+^ protein p97 consists of an N-domain and two tandem ATPase domains D1 and D2, which are connected by the N-D1 and the D1-D2 linkers. Inclusion of the D1-D2 linker, a 22-amino acid peptide, at the end of p97 N-D1 truncate has been shown to activate ATP hydrolysis of its D1-domain, although the mechanism of activation remains unclear. Here, we identify the N-terminal half of this linker, highly conserved from human to fungi, is essential for the ATPase activation. By analyzing available crystal structures, we observed that the D1-D2 linker is capable of inducing asymmetry in subunit association into a p97 hexamer. This observation is reinforced by two new crystal structures, determined in the present work. The effect of D1-D2 linker on the ATPase activity of the D1-domain is correlated to the side-chain conformation of residue R359, a trans-acting arginine-finger residue essential for ATP hydrolysis of the D1-domain. The activation in D1-domain ATPase activity by breaking perfect six-fold symmetry implies functional importance of asymmetric association of p97 subunits, the extent of which can be determined quantitatively by the metric Asymmetric Index.

As one of the most abundant cellular proteins, human p97, also known as VCP (valosin-containing protein), has been assigned an ever-expanding role in a variety of cellular pathways^1^. VCP/p97 is a homo-hexamer with each subunit consisting of one N-terminal domain (N-domain) and two tandem AAA^+^ ATPase domains (D1- and D2-domain)^2^,^3^,^4^. Connecting these domains are two linkers: the N-D1 linker between the N- and D1-domain, and the D1-D2 linker between the D1- and D2-domain (Fig. 1A). Truncates of each individual domain (N-domain or D2-domain) or multiple domains (N-D1 domain) have often been used to study the molecular mechanism of p97 function^3^,^5^,^6^,^7^,^8^,^9^,^10^. Paradoxically, the N-D1 truncate (residues 1–481, N-D1 long or ^ND1^p97^Lng^) used in some studies was shown to retain ~50% of the ATPase activity of full-length p97 (^FL^p97)^6^, whereas in other cases using slightly shorter versions of the N-D1 constructs (residues 1–458^11^ & 1–452^12^, N-D1 short or ^ND1^p97^Shrt^) showed <10% of the full-length ATPase activity. Recently, expressing both the ^ND1^p97^Shrt^ (1–458) and ^ND1^p97^Lng^ (1–480) constructs in parallel, Chou *et al*. revealed that the longer form has 56-fold higher ATPase activity than the short form^13^, providing an explanation for the apparent discrepancy in the observed ATPase activities.

In the structure of ^FL^p97, the D1-domain ends at residue S459 and is followed by the D1-D2 linker (N460-G481), which is fully ordered and has been modeled as a random loop with no apparent secondary structure^3^. Despite differences in the ATP-hydrolyzing abilities of ^ND1^p97^Lng^ and ^ND1^p97^Shrt^, both forms of p97 truncates can be crystallized in various forms and share overall structural features^5^,^10^,^14^. The functional role that this 22-residue D1-D2 linker plays in regulating the ATPase activity of the D1-domain and how it affects the function of p97 are the aims of the current study.

## Results

### The N-terminal half of the D1-D2 linker is critical to the D1-domain activity

**Figure 1:**
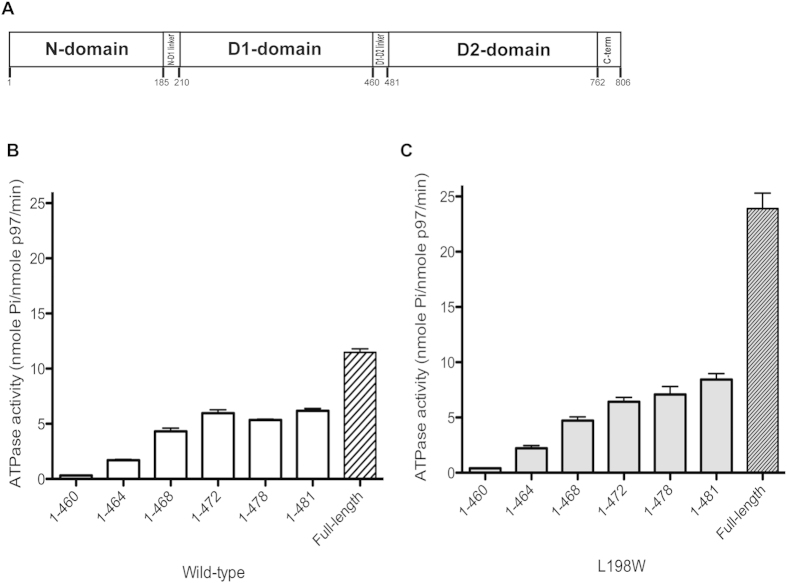
The N-D1 truncates of p97 variants and their ATPase activities. (**A**) Schematic domain organization of p97. (**B**) Specific ATPase activities of wild-type N-D1 truncates in various lengths and wild-type full-length p97. (**C**) Specific ATPase activities of various N-D1 truncates and full-length p97 bearing the L198W mutation. Each data point is an average of at least three independent measurements.

To shed more light on the mechanism of the D1-D2 linker in activating the ATPase activity of the D1-domain and identify the segment that is responsible, we generated a series of N-D1 truncates containing different lengths of the D1-D2 linker, and measured their ATPase activities. Similar to what was reported^13^, the construct without the D1-D2 linker (residues 1–460) retained only 2.7% (0.31 nmole Pi/nmole p97/min) of the ATPase activity of ^FL^p97 (Fig. 1B). However, as more residues of the D1-D2 linker were included, the ATPase activity increased progressively and reached a plateau (~6 nmole Pi/nmole p97/min) in the construct having residues up to P472 (Fig. 1B). This result suggests the presence of the N-terminal half of the D1-D2 linker (residues 460–472) is critical to an active D1-domain. A similar effect can be observed in the IBMPFD (inclusion body myopathy with Paget’s disease of the bone and frontotemporal dementia) mutant L198W (Fig. 1C), indicating the activation of ATPase activity at the D1-domain by the D1-D2 linker is not affected by the introduction of the pathogenic mutation. The higher ATPase activity for the full-length p97 bearing the disease-associated mutation has been characterized earlier^6^.

### The N-terminal half of the D1-D2 linker is highly conserved from eukaryota to archaea

**Figure 2:**
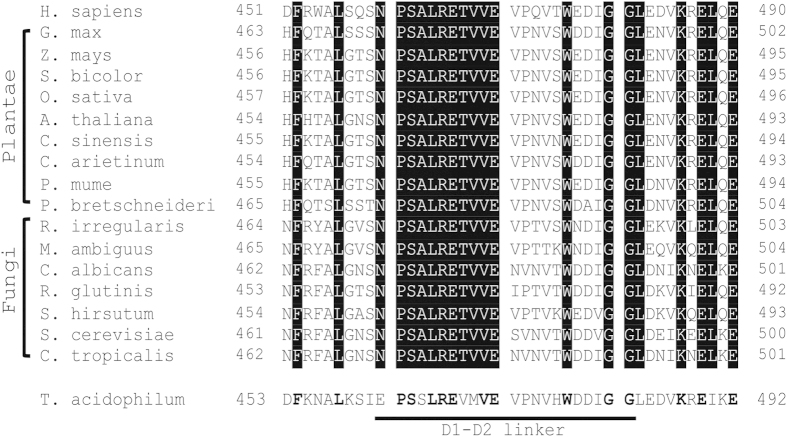
Amino acid sequence alignment of p97 homologs in the D1-D2 linker region. The alignment is based on sequences of *H. sapiens* (NP_009057); *G. max* (XP_003523092); *Z. mays* (XP_008660526); *S. bicolor* (XP_002465842); *O. sativa* (AAP53974); *A. thaliana* (NP_568114); *C. sinensis* (XP_006493028); *C. arietinum* (XP_004487074); *P. mume* (XP_008243978); *P. bretschneideri* (XP_009362967); *R. irregularis* (EXX76046); *M. ambiguus* (GAN06451); *C. albicans* (KHC67689); *R. glutinis* (EGU13311); *S. hirsutum* (XP_007304536); *S. cerevisiae* (NP_010157); *C. tropicalis* (XP_002549770); *T. acidophilum* (WP_010901251). Conserved residues are highlighted.

Amino acid sequences of p97 and its eukaryotic homologues are highly conserved. Pair-wise sequence alignments between human p97 and its homologues including green plants and fungi revealed a sequence identity between 69% and 79%. Multiple sequence alignment across the 16 homologues of p97 listed in Fig. 2, only 51% of the residues (409 residues out of 806 of human p97) are identical. Among the three main domains of p97, the D1-domain (residues 211–459) is the most conserved, with a sequence identity of 69%, followed by the D2-domain (residues 482–762) and the N-domain (residues 1–184) with 51% and 32% sequence identities, respectively. Whereas the N-D1 linker (residues 185–210) shows a 50% sequence identity across different species, the D1-D2 linker (residues 460–481) has a higher sequence identity of 64%. The most striking revelation of the sequence alignment is the degree of conservation of the first 11 residues (residues 460–470) in the D1-D2 linker, which is invariable in our sequence alignment (Fig. 2) and coincides with the maximal activation of ATPase activity of various N-D1 truncates (Fig. 1B,C). This correlation between high sequence conservation and critical role in activation of D1-domain ATPase activity of the D1-D2 linker suggests its indispensable function in p97 over the course of evolution. The sequence conservation is perhaps not limited to eukaryotic organisms because VCP-like ATPase (VAT) in archaeal *T. acidophilum* has an overall sequence identity of 45% to human p97, but the sequence identity for the first 11 residues of the D1-D2 linker remains to be 64% (Fig. 2).

### Location and conformation of the D1-D2 linker

**Figure 3:**
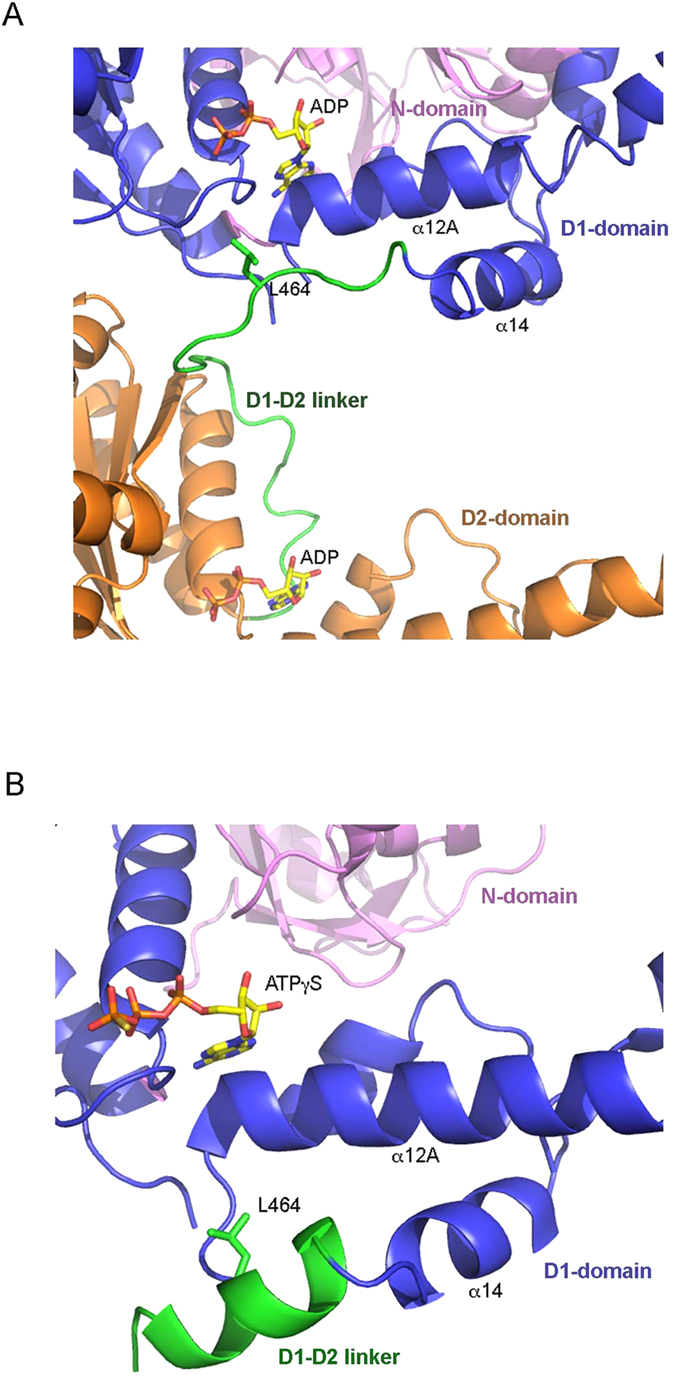
Structures and conformations of the p97 D1-D2 linker. (**A**) Conformation of the D1-D2 linker in the context of full-length p97 structure (PDB:3CF3) with both D1- and D2-domains bound with ADP. The structure is rendered as a cartoon with the N-domain colored in magenta, the D1-domain in blue and the D2-domain in gold. The D1-D2 linker is shown as a green coil. Bound ADP molecules are shown as stick models with carbon in yellow, oxygen in red, nitrogen in blue and phosphorus in orange. Residue L464 in the D1-D2 linker is shown as a stick model in green and is labeled. (**B**) Helical conformation of the D1-D2 linker in the context of a ^ND1^p97^Lng^ structure (PDB:4KO8) with D1-domain bound with ATPγS. Only the N-domain, D1-domain and the D1-D2 linker are shown. The color code is the same as in (**A**).

Among all the published structures of wild-type ^FL^p97, the nucleotide states at the D2-domains vary, bound with either ADP, ADP-AlFx or AMP-PNP, whereas the nucleotides found at the D1-domains are invariably ADP^3^,^4^. In the full-length structures, the D1-D2 linker begins immediately after the last helix (α14) of the D1-domain at residue S459, and has been modeled as a random loop with no well-defined secondary structural elements. The beginning of the D1-D2 linker is found below helix α12A (residues 409–426) of the D1-domain, the loop extends towards the entrance of the D1 nucleotide-binding site and it begins to turn away from the site at residues L464 (Fig. 3A). The D1-D2 linker in a similar conformation can also be observed in the structure of isolated D2-domain that includes the D1-D2 linker (residues 463–806) and forms a heptamer^3^.

In the structures of ^ND1^p97^Lng^ (residues 1–481) with ADP bound (Table 1), the polypeptide chains could only be traced up to residue S462, indicating that the D1-D2 linker is largely disordered, which is consistent with the random loop observed in the structures of ^FL^p97. By contrast, when ATPγS is bound to the D1-domain, a few more residues from the D1-D2 linker could be traced in the electron density but the majority remains disordered. However, in the highest resolution structure containing the R155H mutation and with bound ATPγS (PDB:4KO8 at 1.98 Å resolution), one out of two subunits in the asymmetric unit has the D1-D2 linker traced up to residue V469 (Fig. 3B). Interestingly, instead of a random loop, this D1-D2 linker folds into an α-helix starting from P461. This result is consistent with the secondary structure prediction, suggesting this 10-residue peptide (P461-E470) has a high propensity to form a helix, even though the exact circumstances under which the helix may form remain unclear. Similar to the full-length structures, this helical linker extends below helix α12A of the D1-domain towards the entrance of the ATP-binding pocket of the D1-domain (Fig. 3B).

### The D1-D2 linker prompts asymmetric association of p97 subunits

Reviewing all crystal structures of hexameric p97 available in the Protein Databank (PDB) determined in various lengths, nucleotide states and in the absence of adaptor proteins, we found it interesting that structures of p97 containing the D1-D2 linker were usually determined from crystals with lower symmetry (Table 1). In other words, these structures have multiple subunits in a crystallographic asymmetric unit, which are related by non-crystallographic symmetry (NCS). For example, wild-type ^FL^p97 was crystallized in three different space groups. Two of them contain NCS symmetry (PDB: 3CF1, 3CF2, and 3CF3), except for the structure with the PDB code 1R7R. The latter structure was poorly determined, as was acknowledged by the authors^4^. Likewise, all ^ND1^p97^Lng^ with the D1-D2 linker included were crystallized in crystals of lower symmetry (PDB: 4KOD, 3HU1, 3HU2, 4KLN, and 4KO8). By contrast, the wild-type ^ND1^p97^Shrt^ without the D1-D2 linker (PDB:1E32) was crystallized in a high symmetry crystal system, having a space group of *P*622 with one subunit per crystallographic asymmetric unit. Since all structures of ^ND1^p97^Lng^ also bear single-site pathogenic mutations, this survey seems to indicate two possibilities: one is that when the D1-D2 linker is present, the subunits within a hexameric p97 arrange themselves in a way that deviates from perfect or proper six-fold symmetry, as the linker is absent. We term this asymmetric subunit association or simply asymmetry. Another possibility is disease-associated mutations cause the asymmetry.

To test the above two possibilities, we crystallized wild-type ^ND1^p97^Lng^ with the D1-D2 linker (residues 1–481) in the presence of ADP. This wild-type ^ND1^p97^Lng^ construct crystallized in the low symmetry space group *P*2_1_2_1_2_1_ with 12 subunits per asymmetric unit forming two hexamers (Tables 1 and 2), which is very different from the corresponding short form ^ND1^p97^Shrt^ that crystallized in the high symmetry space group *P*622 (PDB:1E32) (Table 1). Since all previous structures of ^ND1^p97^Lng^ proteins were solved using pathogenic p97 mutants, the solution of this wild-type ^ND1^p97^Lng^ structure has confirmed that the deviation from a perfect hexameric association of the ^ND1^p97^Lng^ is not a consequence of pathogenic mutations but is caused by the D1-D2 linker.

The notion that pathogenic mutations are not the cause of lower symmetry crystals gained further support by the structure of ^ND1^p97^Shrt^ bearing the L198W mutation, determined in the present study with ADP bound (Table 2). During the crystallization of L198W ^ND1^p97^Shrt^ (residues 1–460), AMP-PNP was added. However, due to the instability of AMP-PNP under acidic crystallization conditions, ADP was found instead at the D1-domain in the structure. Consistent with our hypothesis, this pathogenic mutant without the D1-D2 linker crystallized in the space group *P*622, containing a 6-fold crystallographic axis that, just like in the wild-type ^ND1^p97^Shrt^, coincides with the molecular 6-fold axis (Tables 1 and 2).

**Figure 4:**
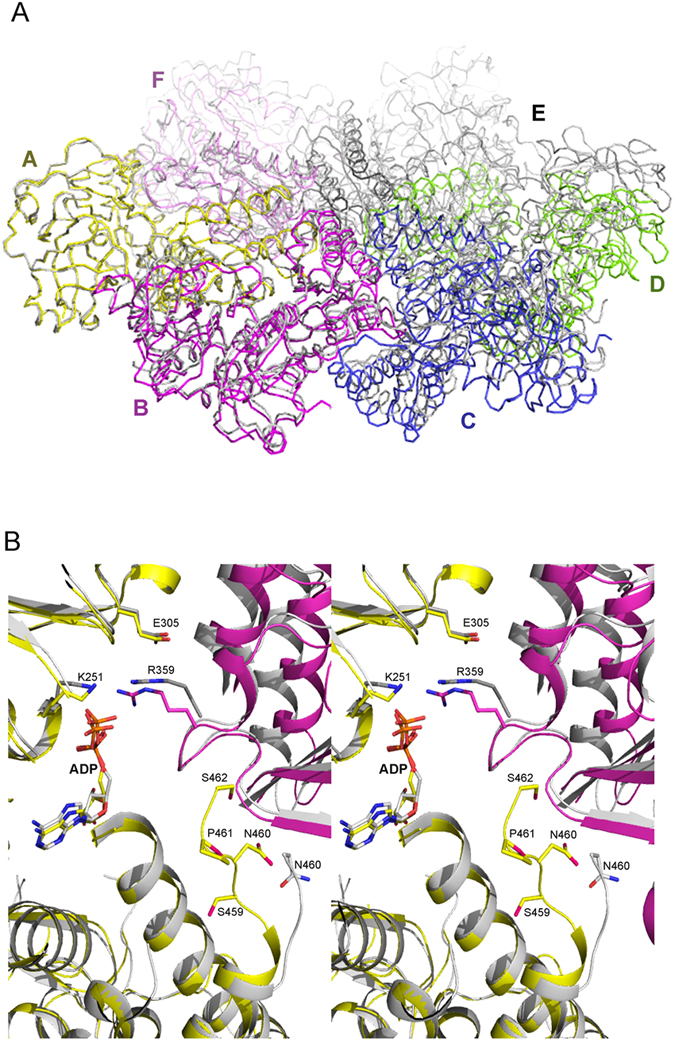
Asymmetry in subunit association revealed by structure alignment. (**A**) Structure superposition of wild-type ^ND1^p97^Shrt^ (PDB:1E32) and wild-type ^ND1^p97^Lng^ (PDB:5DYI) hexamers. Only subunit A from each hexamer was superposed. Subunits of the ^ND1^p97^Lng^ structure are in different colors and labeled. All subunits of ^ND1^p97^Shrt^ are in gray. (**B**) A close-up on the nucleotide-binding interface between subunits A and B from superposition of subunit A of the hexameric structure of L198W ^ND1^p97^Shrt^ in gray (PDB:5DYG) and that of R155H ^ND1^p97^Lng^ in yellow (PDB:4KOD). The adjacent subunit for R155H ^ND1^p97^Lng^ is colored magenta and that for L198W ^ND1^p97^Shrt^ is darker gray. The bound ADP molecules are shown as stick models. Motifs necessary for the ATP cycle such as the Walker A (K251), Walker B (E305Q) and SRH (R359) and the D1-D2 linker observed in R155H ^ND1^p97^Lng^ are shown as stick models and labeled.

To visualize this asymmetry, we used two ADP-bound wild-type p97 N-D1 truncates: ^ND1^p97^Shrt^ (PDB:1E32), determined previously^10^ and ^ND1^p97^Lng^ (PDB:5DYI), determined in this work. A hexameric ring of ^ND1^p97^Shrt^ was constructed by applying crystallographic symmetry operations to the ^ND1^p97^Shrt^ subunit and this hexamer was used to compare with the ^ND1^p97^Lng^ hexamer. We superposed one subunit (subunit A) of the hexameric wild-type ^ND1^p97^Shrt^ with the equivalent subunit in the wild-type hexameric ^ND1^p97^Lng^. Whereas subunits A of the two hexamers align well (rms deviation over 426 residues = 0.65 Å), all the other subunits are out of alignment, as the ^ND1^p97^Lng^ hexamer ring is out of the plane of the ^ND1^p97^Shrt^ ring (Fig. 4A). Although this misalignment does not necessarily mean a lack of proper six-fold symmetry for the ^ND1^p97^Lng^ hexamer, which we shall define more precisely in the next section, it is an indication that the D1-D2 linker alters interactions between neighboring subunits.

### The Asymmetric Index: a measurement for the asymmetric association of p97 subunits

To obtain a more quantitative measurement of the amount of deviation in p97 subunit association from perfect six-fold symmetry in each structure, we devised a metric, asymmetric index (Asym Index in degree, Table 3), that measures the average deviation between near parallel cross vectors that are constructed from vectors connecting equivalent atoms in the structure of a p97 hexamer (see Fig. S1 and Experimental Procedures section for details). Based on this metric, a proper or perfect hexamer should have an Asym Index of 0 degree (0°), because all such cross vectors are parallel to each other and to the six-fold molecular axis. For instance, the structure of the wild-type ^ND1^p97^Shrt^ that crystallized into the space group of *P*622 (PDB:1E32) has an Asym Index of 0°. For structures that crystallized into lower symmetry crystal forms, we selected sequence ranges for residues that have well-defined electron densities from each domain, which were used to calculate the Asym Index for that domain (Table 3). We calculated the Asym Index for five regions: the N-domain, and two each for the AAA ATPase domains (the RecA domain and the helical domain) (Table 3). Based on the calculations, we conclude that the more subunits present in a crystallographic asymmetric unit (more NCS-related subunits), the larger the Asym Index is. This conclusion is consistent with our initial intuitive observation that when the D1-D2 linker is present, there are lower symmetry crystal forms.

Although different domains or sub-domains have different Asym Index values, on average, the ADP-bound forms, for both full-length and N-D1 fragments with the D1-D2 linker, display the greatest deviation from proper six-fold symmetry (Table 3), which is in agreement with previous observations on full-length p97^2^,^3^. The ATPγS-bound forms of ^ND1^p97^Lng^ have, in general, lower Asym Index values but the binding of ATPγS induces more deviation to the N-domain than to other domains. Clearly, this metric provides a quantitative measurement for the observed role of the D1-D2 linker in offsetting each p97 subunit from a proper hexamer.

### Correlation between D1-domain ATPase activity and side-chain mobility of trans-acting arginine-finger residue R359

Interactions between neighboring subunits when the D1-D2 linker is present are quite different (Fig. 4B). To illustrate the differences, we chose the structure of L198W ^ND1^p97^Shrt^ (PDB:5DYG), determined in this work, and that of R155H ^ND1^p97^Lng^ (PDB: 4KOD) for comparison. These two structures have the highest resolutions among the structures with the same N-D1 length; both have ADP bound; they feature the same N-domain conformation; and as we have shown, disease-related mutations do not interfere with subunit association (Table 1). We superposed subunit A of L198W ^ND1^p97^Shrt^ hexamer with equivalent subunit of R155H ^ND1^p97^Lng^. While the structural alignment for subunit A is quite good (rms deviation = 0.78 over 429 residues), there is a significant misalignment observed for the two adjacent subunits they interact with. Here, we focus on the ADP-binding environment for the two hexamers. The C-terminus of ^ND1^p97^Shrt^ has its ends at N460, but a few more residues from the D1-D2 linker can be seen in the ^ND1^p97^Lng^ structure, ending at residue S462 (Fig. 4B). This D1-D2 linker points toward the nucleotide binding pocket of the same subunit and is adjacent to the *in trans* SRH (second region of homology) motif of the neighboring subunit, which is one of the defining features of the family of AAA^+^ proteins, in addition to the Walker A and B motifs^15^. Within this SRH motif, the highly conserved arginine finger (Arg-finger) residue R359 that protrudes from the neighboring subunit and reaches into the nucleotide-binding site to interact, *in trans*, with the bound nucleotide was observed to undergo significant movement. The guanidine group moves on average more than 3 Å when the D1-D2 linker is present (Fig. 4B).

This *in trans* Arg-finger residue R359 is known to undergo large conformational change during the ATP hydrolysis cycle. When the D1-domain is bound with ADP, the guanidine group of R359 is pointing towards the β-phosphate of ADP from the adjacent subunit. When ATPγS occupies the D1-domain, the side-chain of R359 moves out of the way to accommodate the γ-phosphate of the ATPγS (Fig. S2). The side chain mobility of R359 is also clearly influenced by the D1-D2 linker. When the D1-D2 linker is missing, the side chain of R359 is well ordered, interacting with bound ADP in the neighboring subunit, as shown in both ^ND1^p97^Shrt^ structures (Table 1) by well-defined electron density for the side chains (Fig. S3A,B). By contrast, in all structures containing the D1-D2 linker, the electron densities for the side-chain of R359 is absent in all subunits in the asymmetric unit, indicating this residue becomes disordered in the presence of the D1-D2 linker (Fig. S3C–E).

## Discussion

The phenomenon of asymmetry in structures of AAA^+^ proteins and its relation to function has been extensively documented, especially for those with asymmetry induced by binding of nucleotides. A well-studied example is bacterial ClpX, in which it is essential for each subunit in the hexameric assembly to have a different affinity for the nucleotide in order to carry out ATP hydrolysis^16^,^17^. The full-length bacterial unfoldase ClpA with bound ADP assembles into a hexameric spiral structure, deviating from a hexameric ring in solution, suggesting a mixed nucleotide state in solution^18^, and a similar observation was made for bacterial disaggregase ClpB^19^. Dramatic asymmetry has been induced in the bacterial enhancer-binding protein NtrC1 upon partial ATP occupancy^20^.

The structural asymmetry of p97 has been noticed in both full-length p97 and disease-related N-D1 truncates upon interaction with nucleotides^3^,^5^,^6^,^8^,^21^. For wild-type p97, the nucleotide state of each D1-domain controls the movement of its cognate N-domain and is tightly regulated to coordinate multiple nucleotide states among the six chemically identical subunits, resulting in sub-stoichiometric amounts of pre-bound ADP at the D1-ring^6^,^8^,^22^. Even in the presence of saturating amounts of ATPγS, a subset of D1-domains in wild-type p97 is still bound with ADP, leading to the hypothesis that there is an asymmetric conformational movement of N-domains within the hexameric p97^5^,^6^,^23^. This asymmetrical N-domain movement in wild-type p97 seems to be important for its cellular functions, as pathogenic mutants that showed symmetric, or uniformed, movement are functionally defective^5^,^6^. However, how the asymmetry in the D1-domain nucleotide states is established within a homo-hexamer is unknown. In other words, it is not clear what sequence or structural elements in p97 subunits allow each subunit to exist in different nucleotide states.

In this paper, we show that one such element might be the linker connecting the D1- and D2-domain. This linker, when present, activates D1 ATPase activity and simultaneously induces asymmetrical arrangement of the p97 subunits, even though structurally the linker appears to be either disordered in N-D1 fragments or a loop with no defined secondary structures in the full-length protein. Thus, we surmise that asymmetry is required for the activation of the N-D1 ATPase activity and the increase in ATPase activity by breaking the 6-fold symmetry supports the hypothesis that p97 works asymmetrically^5^. In addition to the relationship revealed between the linker and asymmetry, our study has set the stage to understand the role of this linker in regulating D1-domain activity and how the linker may affect the function of full-length p97.

Aside from the difference in ATPase activity, both ^ND1^p97^Shrt^ and ^ND1^p97^Lng^ have similar (within 2-fold) nucleotide-binding affinities, as determined by surface plasmon resonance (SPR)^13^, indicating the low ATPase activity in the ^ND1^p97^Shrt^ is not likely to be due to its inability to bind nucleotides. Indeed, structure superposition of short and long p97 subunits did not show noticeable differences with respect to nucleotide-binding elements such as the Walker A and B motifs (Fig. 4B). In other words, the lack of ^ND1^p97^Shrt^ ATPase activity cannot be attributed to changes in these structural motifs.

Besides the Walker A and B motifs, another highly conserved nucleotide-binding element in AAA^+^ proteins is the SRH (second region of homology) motif. In a classic study on the bacterial AAA^+^ protein FtsH by Karata *et al*., mutations were introduced to this motif, which led to the loss of ATPase activity without significant impact on the binding of nucleotide^15^. In p97, the SRH motif in the D1-domain is 346-ATNRPNSIDPALR**R**FG**R**FD-364, which contains two perfectly conserved arginine residues, R359 and R362. Crystal structures of both full-length p97 and N-D1 fragments showed that R359 extends its side chain into the nucleotide-binding site of an adjacent subunit, interacting directly with the bound nucleotide, which qualifies it to be an *in trans* Arg-finger residue. Alanine substitution of R359 in N-D1 truncated p97 altered the hexameric status of the protein, resulting in complete loss of ATP hydrolysis^24^. In full-length p97, the R359 mutation did not have any effect on the nucleotide binding in the D2-domain, as determined by the tryptophan fluorescence spectrum and by isothermal titration calorimetry (ITC), but displayed a severe reduction (90%) in ATPase activity^24^. These mutational studies demonstrated that changes to the R359 residue led to loss of ATPase activity in the D1-domain.

In the present study, we have provided structural evidence to show that conformational alterations to the side chain of the trans-acting Arg-finger residue R359 forms the structural basis for the observed activation of D1 ATPase activity by the D1-D2 linker. To reiterate, the multi-structure alignment of ^ND1^p97^Shrt^ with ^ND1^p97^Lng^ and ^FL^p97, all in the ADP bound form, showed significant misalignment of the adjacent subunits leading to a side chain displacement of R359 that cannot be ignored (>3 Å) (Fig. 4B). This displacement appears to stabilize the side-chain of R359 in ^ND1^p97^Shrt^, which is disordered in both ^ND1^p97^Lng^ and ^FL^p97 (Fig. S3), by interacting strongly with the bound nucleotide, resulting in a symmetric arrangement of p97 that is defective in hydrolyzing ATP.

Although able to participate in various cellular pathways, p97 has been considered, by a emerging consensus, to perform the function of protein segregase, disrupting protein complexes or extracting proteins from the membrane environment^1^. With 12 ATPase domains stacked in two separate rings, it is conceivable that there are communication channels among these ATPase domains, in particular between the two rings. Indeed, evidence of communication between the D1 and D2 rings of p97 has been accumulating. Binding of nucleotide at the D1-domain is required for the ATPase activity of the D2-domain^6^,^12^; and a motion transmission from D2-domain as a result of nucleotide or ligand binding to the D1-domain is mediated through the D1-D2 linker^2^,^3^,^21^,^25^. A conformational change in the D1-D2 linker may therefore serve as a means of communication between the two ATPase domains of p97. However, our current finding does not provide evidence if such communication channel is inter-protomer as proposed by Li *et al*.^25^, or intra-protomer. Since the D1-D2 linker bridges the two domains, its structure could be changed in response to changes in both domains, such as changes resulting from nucleotide binding. Although the linker is proposed to serve as a “lever” to pry changes in the D1-domain, changes in the nucleotide state at the D2-domain do not seem to affect the secondary structure of the D1-D2 linker, as it always appears to be a loop regardless of the nucleotide state at the D2-domain^2^,^3^. How changes in the D1-domain might affect the conformation of the D1-D2 linker in the context of full-length p97 is not clear, because in all available full-length p97 structures the D1-domains are always bound with ADP. Nevertheless, studies with N-D1 fragments did provide a hint that the D1-D2 linker may undergo nucleotide-dependent secondary structure change going from random loop in the presence of ADP to a helical segment in the presence of ATPγS (PDB: 4KO8), which is consistent with the high helical propensity of this linker from secondary structure prediction. Although the exact circumstances under which the helical secondary structure may form for the D1-D2 linker remain to be seen, nucleotide-induced loop-to-helix conformational change has been observed in the N-D1 linker of p97^5^. The N-D1 linker is a random loop when the D1-domain is occupied by ADP and becomes a helix when bound with ATPγS, thus driving the N-domain to undergo both rotational and translational conformational change.

## Experimental Procedures

### Materials

All chemicals were sourced from Sigma Aldrich (St. Lois, MO) unless otherwise stated.

### Plasmids construction, protein expression and purification of p97 N-D1 variants

Expression and purification of p97 was done as previously described^5^. Variants of p97 N-D1 containing different lengths of the D1-D2 linker were generated using a QuikChange Site-Directed Mutagenesis kit (Agilent Technologies, Santa Clara, CA).

### Determination of ATPase activity

ATPase activity of p97 was determined by measuring the amount of inorganic phosphate released from ATP hydrolysis, which reacts with a complex of molybdate and malachite green^26^,^27^. The activity assay was performed in an assay buffer containing 50 mM Tris-HCl, pH 8.0, 20 mM MgCl_2_, 1 mM EDTA, and 1 mM DTT. A total of 50 μl reaction mix containing 2–5 μg of protein and 4 mM ATP in the assay buffer was incubated at 37 °C for 15 min. The reaction was immediately stopped by the addition of 800 μl dye buffer (a fresh mixture of 0.045% malachite green and 1.4% ammonium molybdate tetrahydrate in 4 N HCl in a 1:3 ratio) followed by the addition of 100 μl of 34% sodium citrate solution after 1 min. After 10 min incubation at room temperature, 16 μl of 10% Tween-20 was added to dissolve any precipitate. Absorbance was then measured at 660 nm. The amount of inorganic phosphate released was calculated based on the standard curve established by a known amount of KH_2_PO_4_ (50–300 μM) in assay buffer.

### Crystallization and structure determination

Crystals of p97 N-D1 truncates were grown using the sitting-drop vapor diffusion method at 16 °C. A protein solution of 7 mg/ml was mixed with MgCl_2_ and ADP or AMP-PNP to a final concentration of 40 mM and 4 mM, respectively, and incubated on ice for 30 min before spun at 14,000 rpm for 30 m^28^ in at 4 °C. The admixture of 2 μl wild-type ^ND1^p97^Lng^ and an equal volume of a well solution containing 0.1 M sodium citrate, pH 6.0, 0.3 M NaCl, 15.2% PEG3350, 20% glycerol, 2% benzamidine was set up for crystallization. Crystals were cryo-protected with the well solution supplemented with 20% glycerol and flash-frozen in liquid propane. Similarly, crystals of L198W ^ND1^p97^Shrt^ were grown by mixing 1:1 with a well solution containing 3.7 M sodium formate, pH 6.0, 8% glycerol. Crystals were cryo-protected with the well solution supplemented with 25% 7 M sodium formate and flash-frozen in liquid propane.

X-ray diffraction experiments were carried out at 100 K at the SER-CAT beam lines of the Advanced Photon Source at Argonne National Laboratory. Diffraction images were collected with MarCCD detectors, and processed and scaled with the HKL200 package^29^. The diffraction data for wild-type ^ND1^p97^Lng^ was anisotropic, the scaled data was truncated and scaled with the diffraction anisotropy server (http://services.mbi.ucla.edu/anisoscale)^30^. The resulting data was then used for structural determination. The structures were determined by molecular replacement using PDB:4KOD^5^ as a search model using the program MOLREP^31^ in CCP4 program package^32^. The structures were refined using Refmac^33^ in the CCP4 package. All structure models were manually built using the program COOT^34^.

### Calculation of Asym Index

For a proper hexamer, the i^th^ atom of subunit j ***x***_i,j_ (*x*_i,j_, *y*_i,j_, *z*_i,j_) is related by a six-fold symmetry axis with an equivalent atom in another subunit k ***x***_i,k_ (*x*_i,k_, *y*_i,k_, *z*_i,k_). Here, i goes from 1 to N, N is the number of atoms in the subunit; j and k take the number from 1 to 6 and j does not equal to k. For each atom ***x***_i,j_, a vector (***a***_i,j_) can be drawn such that ***a***_i,j_ = ***x***_i,j_ − ***x***_*i,j*+1_, (*j* = 1, 2, … and 6 for a hexamer and *j* + 1 ≤ 6). Here ***x***_*i,j*+1_ is the equivalent atom from a neighboring subunit. Correspondingly, a cross vector ***b***_i,k_ is calculated between the two neighboring vectors ***b***_i,k_ = ***a***_i,k_ **x** ***a***_i,k+1_, where *k* = 1, 2, …, and 6. For a perfect or proper hexamer, the cross vectors ***b***_i,k_ are all parallel to each other, whereas for a hexamer that deviates from a perfect hexamer, the cross vectors may not be parallel. Thus, the asymmetric index (Asym Index) is defined as the average angle in degrees (°) among pairs of cross vectors:


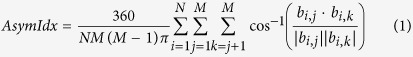


## Additional Information

**Accession codes**: Atomic coordinates and structure factors have been deposited in the Protein Data Bank, under the accession codes 5DYI for the structure of WT ND1p97Lng and 5DYG for the structure of L198W ND1p97Shrt.

**How to cite this article**: Tang, W. K. and Xia, D. Role of the D1-D2 Linker of Human VCP/p97 in the Asymmetry and ATPase Activity of the D1-domain. *Sci. Rep.* **6**, 20037; doi: 10.1038/srep20037 (2016).

## Supplementary Material

Supplementary Information

## Figures and Tables

**Table 1:** Correlation between crystal system and full-length, long- and short-form of N-D1 truncates of p97.

**p97 construct**	**Residue range (in structure)**	**Nucleotide D1/D2**	**Space group (No.)**	**PDB**	**No. sub/AU**	**Cell dimension (*****a, b, c, α, β, γ***)	**Res (Å)**
WT ^FL^p97	1–806 (21–763)	ADP/ADP-AlFx	*I*222 (23)	3CF1^3^	3	162.7 178.0 321.1 90.0 90.0 90.0	4.40
WT ^FL^p97	1–806 (21–763)	ADP/ADP	*I*222 (23)	3CF3^3^	3	164.0 178.9 320.6 90.0 90.0 90.0	4.25
WT ^FL^p97	1–806 (21–759)	ADP/AMP-PNP	*P*3 (143)	3CF2^3^	4	144.9 144.9 164.4 90.0 90.0 120.0	3.50
WT ^FL^p97^a^	1–806 (17–735)	ADP/–	*P*622 (177)	1R7R^4^	1	145.2 145.2 167.4 90.0 90.0 120.0	3.60
WT ^ND1^p97^Shrt^	1–458 (21–458)	ADP/	*P*622 (177)	1E32^10^	1	146.0 146.0 84.7 90.0 90.0 120.0	2.90
L198W ^ND1^p97^Shrt^	1–460 (15–460)	ADP/	*P622* (177)	5DYG^b^	1	145.6 145.6 84.0 90.0 90.0 120.0	2.20
WT ^ND1^p97^Lng^	1–481 (21–461)	ADP/	*P*2_1_2_1_2_1_ (19)	5DYI^b^	12	146.5 176.3 257.0 90.0 90.0 90.0	3.71
R155H ^ND1^p97^Lng^	1–481 (18–462)	ADP/	*P*2_1_2_1_2_1_ (19)	4KOD^6^	12	146.5 170.7 256.6 90.0 90.0 90.0	2.96
R86A ^ND1^p97^Lng^	1–481 (12–463)	ATPγS/	*P*1 (1)	3HU2^5^	6	90.9 102.6 107.2 97.5 90.6 91.5	2.85
R95G ^ND1^p97^Lng^	1–481 (12–462)	ATPγS/	*P*1 (1)	3HU1^5^	6	92.8 103.3 107.7 97.7 91.9 89.8	2.81
A232E ^ND1^p97^Lng^	1–481 (12–460)	ATPγS/	*P*1 (1)	4KLN^6^	6	91.2 104.5 109.5 98.1 90.6 92.7	2.62
R155H ^ND1^p97^Lng^	1–481 (14–469)	ATPγS/	*H*3 (146)	4KO8^6^	2	134.2 134.2 182.9 90.0 90.0 120.0	1.98

^a^Reflection file is not available in PDB.

^b^Structures determined in this work.

**Table 2:** Statistics on the qualities of diffraction data sets and atomic models.

	**WT** ^**ND1**^**p97**^**Lng**^	**L198W** ^**ND1**^**p97**^**Shrt**^
Bound nucleotide	ADP	ADP
Data collection		
Space group	*P*2_1_2_1_2_1_	*P*622
Unit cell [*a, b, c,* Å]	*146.5, 176.3, 257.0*	*145.6, 145.6, 84.0*
[α, β, γ, °]	*90, 90, 90*	*90, 90, 120*
Resolution (last shell) [Å]	50–3.71 (3.84–3.71)	50–2.20 (2.28–2.20)
R_merge_^a^ (last shell) [%]	10.6 (39.8)	9.1 (28.4)
Completeness (last shell) [%]	88.8 (71.9)^c^	97.0 (87.0)
Total observations	306,694	157,021
Unique reflections	62,997	26,399
I/σ(I) (last shell)	7.1 (1.9)	16.3 (3.9)
Refinement statistics		
Resolution [Å]	3.71	2.20
R_free_ (last shell) [%]	28.5 (57.0)	25.7 (36.7)
R_work_ (last shell) [%]	24.9 (46.3)	20.0 (30.7)
Rmsd bond length [Å]	0.012	0.015
Rmsd bond angle [°]	1.635	1.902
Coordinate error (Rfree, Å)	1.16	0.22
Number of non-H atoms atomsatomsatoms	41579	3642
Number of residues	5265	446
Number of solvent molecules	0	97
Number of ATPγS/ADP	12	1
Number of Mg^2+^ ions	0	0
Ramachandran analysis		
Most favored [%]	88.8	91.9
Allowed [%]	11.2	7.9
Generously allowed (%)	0.0	0.3
Disallowed [%]	0.0	0.0
PDB code	5DYI	5DYG

^a^R_merge_ is defined as Σ|**I**_**h,i**_ − <**I**_**h**_>|/Σ**I**_**h,i**_, where **I**_**h,i**_ is the intensity for **i**^th^ observation of a reflection with Miller index **h**, and <**I**_**h**_> is the mean intensity for all measured **I**_**h**_s and Friedel pairs.

^b^Values in parentheses are for the highest resolution shells.

^c^Data was diffracted anisotrpically to a resolution of 3.7 Å in the a* and c* directions and 5.2 Å in the b* direction according to an I/σI > 3 criterion. Data was anisotropically truncated and scaled with the diffraction anisotropy server (http://services.mbi.ucla.edu/anisoscale/). The resulting data set is 68.1% complete overall to 3.7 Å resolution.

**Table 3:** Asymmetric Index values for various p97 structural domains.

**p97 construct**	**Space Group**	**PDB code**	**No. sub per AU**	**Nucleotide D1/D2**	**Asym Index (°)**^a^				
					**N-domain**	**D1-RecA**	**D1-helical**	**D2-RecA**	**D2-helical**
WT ^FL^p97	*I*222	3CF1^3^	3^b^	ADP/ADP•AlF_3_	1.95	1.96	2.13	1.38	0.67
WT ^FL^p97	*I*222	3CF3^3^	3^b^	ADP/ADP	3.48	2.27	1.92	1.38	0.91
WT ^FL^p97	*P*3	3CF2^3^	4^b^,^c^	ADP/AMP-PNP	0.11	0.93	0.40	0.65	0.43
				ADP/AMP-PNP	0.09	1.04	0.34	0.73	0.38
WT ^ND1^p97^Shrt^	*P*622	1E32^10^	1^b^	ADP/–	0.0	0.0	0.0	0.0	0.0
L198W ^ND1^p97^Shrt^	*P622*	5DYG	1^b^	ADP/–	0.0	0.0	0.0	0.0	0.0
WT ^ND1^p97^Lng^	*P*2_1_2_1_2_1_	5DYI	12^c^	ADP/–	4.60	5.81	5.29	–	–
				ADP/–	6.08	6.34	7.45	–	–
R155H ^ND1^p97^Lng^	*P*2_1_2_1_2_1_	4KOD^6^	12^c^	ADP/–	5.71	7.41	7.72	–	–
				ADP/–	4.09	7.50	4.86	–	–
R86A ^ND1^p97^Lng^	*P*1	3HU2^5^	6	ATPγS/–	1.36	0.72	1.19	–	–
R95G ^ND1^p97^Lng^	*P*1	3HU1^5^	6	ATPγS/–	1.09	0.70	1.04	–	–
A232E ^ND1^p97^Lng^	*P*1	4KLN^6^	6	ATPγS/–	2.47	1.22	1.72	–	–
R155H ^ND1^p97^Lng^	*H*3	4KO8^6^	2^b^	ATPγS/–	1.24	1.50	0.61	–	–

^a^Residue ranges used in Asymmetric Index calculation for each domains are: N-domain (24–184), D1-RecA sub-domain (209–369), D1-helical subdomain (374–456), D2-RecA sub-domain (483–586), and D2-helical sub-domain (647–703). Only CA atoms are used for the calculation.

^b^Hexamers were constructed by applying crystallographic symmetry before computing Asym Index.

^c^In these crystals, there are two p97 hexamers in a crystallographic asymmetric unit.

## References

[b1] Meyer H. & Weihl C. C. The VCP/p97 system at a glance: connecting cellular function to disease pathogenesis. J Cell Sci 127, 3877–83 (2014). 25146396 10.1242/jcs.093831 PMC4163641

[b2] DeLaBarre B. & Brunger A. T. Complete structure of p97/valosin-containing protein reveals communication between nucleotide domains. Nat Struc Biol 10, 856–863 (2003). 10.1038/nsb972 12949490

[b3] Davies J. M., Brunger A. T. & Weis W. I. Improved Structures of Full-Length p97, an AAA ATPase: Implications for Mechanisms of Nucleotide-Dependent Conformational Change. Structure 16, 715–26 (2008). 18462676 10.1016/j.str.2008.02.010

[b4] Huyton T. . The crystal structure of murine p97/VCP at 3.6 Å. J Struc Biol 144, 337–348 (2003). 10.1016/j.jsb.2003.10.007 14643202

[b5] Tang W. K. . A novel ATP-dependent conformation in p97 N-D1 fragment revealed by crystal structures of disease-related mutants. Embo J 29, 2217–29 (2010). 20512113 10.1038/emboj.2010.104 PMC2905243

[b6] Tang W. K. & Xia D. Altered intersubunit communication is the molecular basis for functional defects of pathogenic p97 mutants. J Biol Chem 288, 36624–35 (2013). 24196964 10.1074/jbc.M113.488924 PMC3868774

[b7] Hanzelmann P. & Schindelin H. The structural and functional basis of the p97/valosin-containing protein (VCP)-interacting motif (VIM): mutually exclusive binding of cofactors to the N-terminal domain of p97. J Biol Chem 286, 38679–90 (2011). 21914798 10.1074/jbc.M111.274506 PMC3207442

[b8] Davies J. M., Tsuruta H., May A. P. & Weis W. I. Conformational changes of p97 during nucleotide hydrolysis determined by small-angle X-Ray scattering. Structure 13, 183–95 (2005). 15698563 10.1016/j.str.2004.11.014

[b9] Dreveny I. . Structural basis of the interaction between the AAA ATPase p97/VCP and its adaptor protein p47. Embo J 23, 1030–9 (2004). 14988733 10.1038/sj.emboj.7600139 PMC380986

[b10] Zhang X. . Structure of the AAA ATPase p97. Mol Cell 6, 1473–1484 (2000). 11163219 10.1016/s1097-2765(00)00143-x

[b11] Song C., Wang Q. & Li C. C. ATPase activity of p97-valosin-containing protein (VCP). D2 mediates the major enzyme activity, and D1 contributes to the heat-induced activity. J Biol Chem 278, 3648–55 (2003). 12446676 10.1074/jbc.M208422200

[b12] Ye Y., Meyer H. H. & Rapoport T. A. Function of the p97-Ufd1-Npl4 complex in retrotranslocation from the ER to the cytosol: dual recognition of nonubiquitinated polypeptide segments and polyubiquitin chains. J Cell Biol 162, 71–84 (2003). 12847084 10.1083/jcb.200302169 PMC2172719

[b13] Chou T. F. . Specific inhibition of p97/VCP ATPase and kinetic analysis demonstrate interaction between D1 and D2 ATPase domains. J Mol Biol 426, 2886–99 (2014). 24878061 10.1016/j.jmb.2014.05.022 PMC4102644

[b14] Tang W. K. & Xia D. IBMPFD and p97, the structural and molecular basis for functional disruption. in Neuromuscular Disorders 155–174 (InTech, 2012).23200905

[b15] Karata K., Inagawa T., Wilkinson A. J., Tatsuta T. & Ogura T. Dissecting the role of a conserved motif (the second region of homology) in the AAA family of ATPases. Site-directed mutagenesis of the ATP-dependent protease FtsH. Journal of Biological Chemistry 274, 26225–26232 (1999). 10473576 10.1074/jbc.274.37.26225

[b16] Stinson B. M., Baytshtok V., Schmitz K. R., Baker T. A. & Sauer R. T. Subunit asymmetry and roles of conformational switching in the hexameric AAA+ ring of ClpX. Nat Struct Mol Biol 22, 411–6 (2015). 25866879 10.1038/nsmb.3012 PMC4424054

[b17] Glynn S. E., Martin A., Nager A. R., Baker T. A. & Sauer R. T. Structures of asymmetric ClpX hexamers reveal nucleotide-dependent motions in a AAA+ protein-unfolding machine. Cell 139, 744–56 (2009). 19914167 10.1016/j.cell.2009.09.034 PMC2778613

[b18] Guo F., Maurizi M. R., Esser L. & Xia D. Crystal structure of ClpA, an HSP100 chaperone and regulator of ClpAP protease. J Biol Chem 277, 46743–46752 (2002). 12205096 10.1074/jbc.M207796200

[b19] Lee S. . The structure of ClpB: a molecular chaperone that rescues proteins from an aggregated state. Cell 115, 229–240 (2003). 14567920 10.1016/s0092-8674(03)00807-9

[b20] Sysoeva T. A., Chowdhury S., Guo L. & Nixon B. T. Nucleotide-induced asymmetry within ATPase activator ring drives sigma54-RNAP interaction and ATP hydrolysis. Genes Dev 27, 2500–11 (2013). 24240239 10.1101/gad.229385.113 PMC3841738

[b21] DeLaBarre B. & Brunger A. T. Nucleotide dependent motion and mechanism of action of p97/VCP. J Mol Biol 347, 437–452 (2005). 15740751 10.1016/j.jmb.2005.01.060

[b22] Briggs L. C. . Analysis of nucleotide binding to P97 reveals the properties of a tandem AAA hexameric ATPase. J Biol Chem 283, 13745–52 (2008). 18332143 10.1074/jbc.M709632200 PMC2376215

[b23] Tang W. K. & Xia D. Structural and functional deviations in disease-associated p97 mutants. J Struct Biol 179, 83–92 (2012). 22579784 10.1016/j.jsb.2012.04.024 PMC4788498

[b24] Wang Q. . Multifunctional roles of the conserved Arg residues in the second region of homology of p97/valosin-containing protein. J Biol Chem 280, 40515–23 (2005). 16216872 10.1074/jbc.M509636200

[b25] Li G., Huang C., Zhao G. & Lennarz W. J. Interprotomer motion-transmission mechanism for the hexameric AAA ATPase p97. Proc Natl Acad Sci USA 109, 3737–41 (2012). 22355145 10.1073/pnas.1200255109 PMC3309748

[b26] Hess H. H. & Derr J. E. Assay of inorganic and organic phosphorus in the 0.1-5 nanomole range. Anal Biochem 63, 607–13 (1975). 1122033 10.1016/0003-2697(75)90388-7

[b27] Lanzetta P. A., Alvarez L. J., Reinach P. S. & Candia O. A. An improved assay for nanomole amounts of inorganic phosphate. Anal Biochem 100, 95–7 (1979). 161695 10.1016/0003-2697(79)90115-5

[b28] DeLaBarre B. & Brunger A. T. Complete structure of p97/valosin-containing protein reveals communication between nucleotide domains. Nat Struct Biol 10, 856–63 (2003). 12949490 10.1038/nsb972

[b29] Otwinowski Z. & Minor W. Processing of X-ray Diffraction Data Collected in Oscillation Mode. Methods in Enzymology 276, 307–326 (1997). 10.1016/S0076-6879(97)76066-X 27754618

[b30] Strong M. . Toward the structural genomics of complexes: crystal structure of a PE/PPE protein complex from Mycobacterium tuberculosis. Proc Natl Acad Sci USA 103, 8060–5 (2006). 16690741 10.1073/pnas.0602606103 PMC1472429

[b31] Vagin A. & Teplyakov A. MOLREP: an automated program for molecular replacement. Journal of Applied Crystallography 30, 1022–1025 (1997).

[b32] CCP4. Collaborative Computational Project, Number 4, 1994. The CCP4 Suit: Programs for Protein Crystallography. Acta Crystallographica D50, 760–763 (1994).

[b33] Murshudov G. N., Vagin A. A. & Dodson E. J. Refinement of Macromolecular Structures by the Maximum-likelihood Method. Acta Crystallographica D53, 240–255 (1997). 10.1107/S0907444996012255 15299926

[b34] Emsley P. & Cowtan K. Coot: model-building tools for molecular graphics. Acta Cryst. D60, 2126–2132 (2006). 10.1107/S0907444904019158 15572765

