# Supplementary material for: Role of the D1-D2 Linker of Human VCP/p97 in the Asymmetry and ATPase Activity of the D1-domain
Source: Sci Rep. 2016 Jan 28;6:20037. doi: 10.1038/srep20037 (PMC4730245; doi:10.1038/srep20037)
Supplement: Supplementary Information [file srep20037-s1.pdf]

Supplementary Information for

Role of the D1-D2 Linker of Human VCP/p97 in the Asymmetry and ATPase Activity of the D1-domain

Wai Kwan Tang and Di Xia[¶].

Laboratory of Cell Biology, Center for Cancer Research, National Cancer Institute, National Institutes of Health, Bethesda, MD 20892, USA.

[¶]Correspondence should be addressed to DX: e-mail: xiad@mail.nih.gov

Laboratory of Cell Biology, NCI, NIH

37 Convent Drive, Building 37, Room 2122C

Bethesda, MD 20892

Tel: (301)-435-6315

Fax: (301)-480-2315

Supplemental Information

Figure S1. Schematic drawing of vectors defined for the calculation of asymmetric index. Symmetric objects such as the six subunits of p97 are represented by shaded triangles. Atom i (i = 1, 2,…, N), from jth subunit (j = 1,…, 6) is represented by a dot labeled with $\boldsymbol{x}_{i,j}$ in black color. Vectors drawn between pairs of neighboring atoms are shown as blue arrows labeled with $\boldsymbol{a}_{i,j}$. Cross vectors constructed from neighboring vectors are shown as red arrows labeled with $\boldsymbol{b}_{i,j}$. The asymmetric index (Asym Index) is defined as the averaged angle in degree (^{o}) among pairs of cross vectors. For a perfect, proper hexamer, in which all cross vectors are parallel to each other, the Asym Index is zero.

Figure S2. Side-chain conformations of the *in trans* Arg-finger residue R359 in structures of N-D1 p97 bound with either ADP or ATPγS Structures of wild-type ^{ND1}p97^{Shrt} bound with ADP (PDB:1E32) colored in magenta and R155H ^{ND1}p97^{Lng} bound with ATPγS (PDB: 4KO8) in gray are superposed based on the D1-domain. Side-chains of Arg-finger residue R359 and the bound nucleotides are shown in stick representations.

Figure S3. Mobility change revealed for side-chain of the *in trans* Arg-finger residue R359 in ^{ND1}p97^{Shrt} and ^{ND1}p97^{Lng} Side-chains of the *in trans* Arg-finger residue R359 in stick model from four different ADP-bound structures are overlaid with electron density meshes contoured at 1σ level from their respective *2Fo-Fc* maps. The

stick models in magenta, cyan, yellow, green and orange are from structures of (A) wild-type $^{ND1}p97^{Shrt}$, (B) L198W $^{ND1}p97^{Shrt}$, (C) wild-type $^{ND1}p97^{Lng}$, (D) R155H $^{ND1}p97^{Lng}$ and (E) $^{Full}p97$ (PDB:3CF2) respectively.

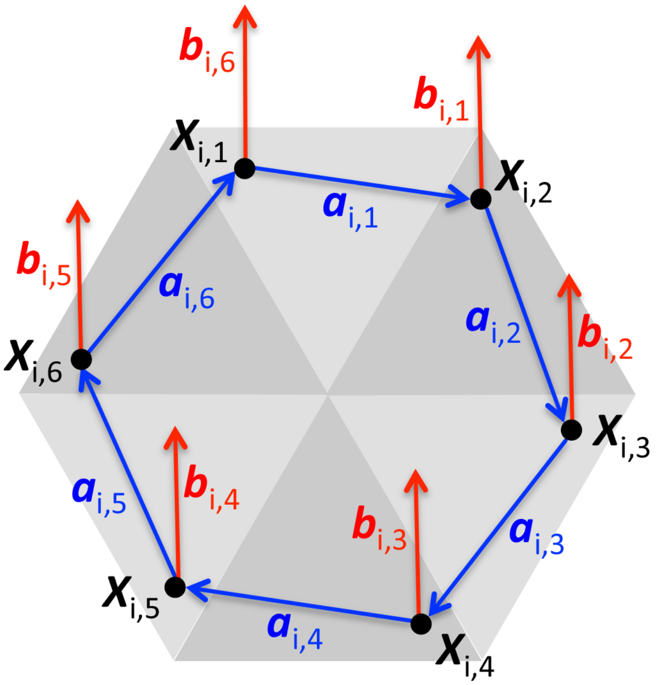

Tang & Xia, Figure.S1

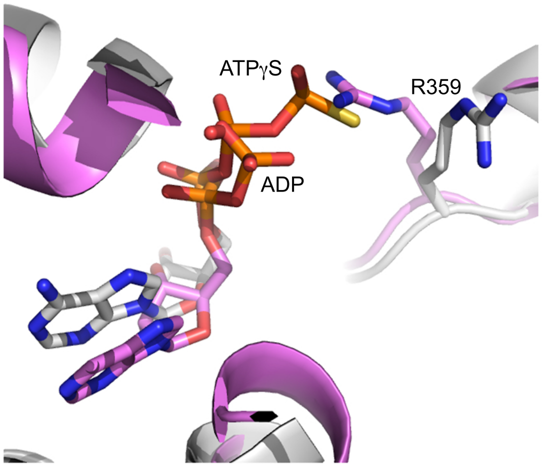

Tang & Xia, Figure S2

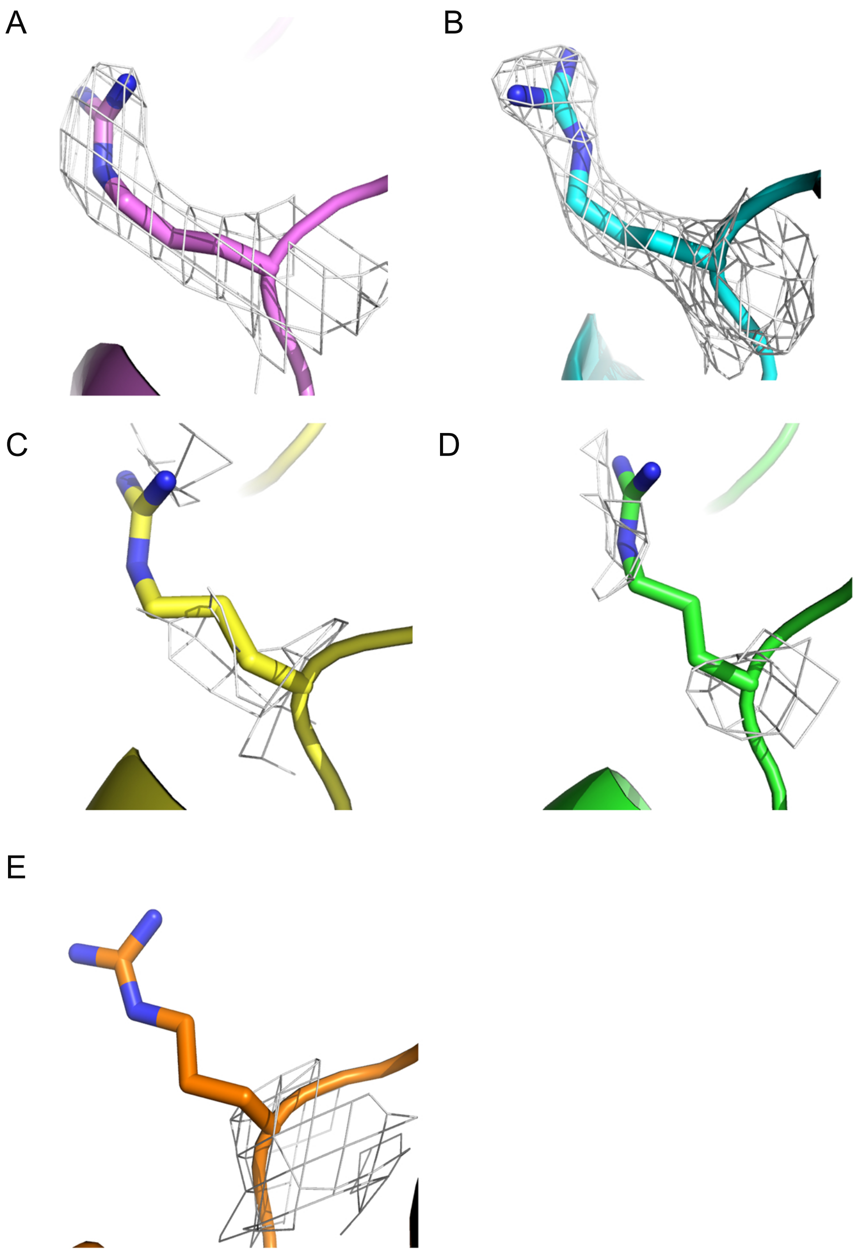

Tang & Xia, Figure S3
